# Prevalence, awareness, treatment, and control of dyslipidemia in Chinese adults: a systematic review and meta-analysis

**DOI:** 10.3389/fcvm.2023.1186330

**Published:** 2023-07-05

**Authors:** Qianhang Xia, Yuquan Chen, Zijing Yu, Zhongyue Huang, Yujie Yang, Ayan Mao, Wuqi Qiu

**Affiliations:** ^1^ Institute of Medical Information/Library, Chinese Academy of Medical Sciences, Beijing, China; ^2^ Peking Union Medical College, Beijing, China

**Keywords:** dyslipidemia, prevalence, awareness rate, treatment rate, control rate, Mainland China, meta-analysis

## Abstract

**Background:**

Researchers have conducted a considerable number of epidemiological studies on dyslipidemia in China over recent years. Nevertheless, a representative study to comprehensively appraise for the epidemiological status of dyslipidemia is still lacked. This meta-analysis is intended to explore the pooled prevalence, rates of awareness, treatment, and control of dyslipidemia among adults in Chinese Mainland.

**Materials and methods:**

A systematic review was performed on relevant cross-sectional studies published since January 2012 by searching six authoritative literature databases. Meta-analyses were conducted in included studies based on a random-effect model to summarize the epidemiological status of dyslipidemia in China. A potential source of heterogeneity was detected by subgroup analysis and meta-regression. Publication bias was assessed by *Egger*'s test and funnel plots. A sensitivity analysis was conducted to examine the study quality's influence on the pooled estimate of prevalence and rates of awareness, treatment, and control.

**Results:**

Forty-one original researches with a total of 1,310,402 Chinese participants were finally included in the meta-analysis. The prevalence, rates of awareness, treatment, and control of dyslipidemia were 42.1%, 18.2%, 11.6%, and 5.4%, respectively. With a pooled prevalence estimate at 24.5%, low HDL-C was the most prevalent among various dyslipidemia types, followed by hypertriglyceridemia (TG) (15.4%), hypercholesterolemia (TC) (8.3%), and high LDL-C (7.1%). The pooled prevalence of elevated serum lipoprotein(a) [Lp(a)] was 19.4%. By gender, the prevalence of dyslipidemia was 47.3% in males and 38.8% in females. Subgroup analyses revealed that the prevalence in southern and urban areas were higher than their counterparts. Females and population in urban areas tended to possess higher rates of awareness, treatment, and control. Meta-regression analyses suggested that the year of screening influenced prevalence estimates for dyslipidemia. The impact of the study's quality on the pooled estimates is insignificant.

**Conclusion:**

Our study suggested a severe epidemic situation of dyslipidemia among adults in Chinese Mainland. More importantly, the awareness, treatment, and control rates were extremely low, revealing that dyslipidemia is a grave health issue. Consequently, we should attach more importance to the management of dyslipidemia, especially in economically underdeveloped areas.

**Systematic review registration:**

PROSPERO [CRD42022366456].

## 1. Introduction

Cardiovascular disease (CVD) is one of the prior sources of global disease burden, as well as the main cause of premature death for Chinese residents (1–3). In China, cardiovascular disease is the leading cause of total deaths among residents while its prevalence and mortality are still increasing (4, 5). In 2019, cardiovascular diseases were responsible for 46.76% of the total deaths in rural areas and 44.26% in urban areas of China (6), which means that approximately two in five deaths were caused by cardiovascular diseases on average. Against the background of population aging trend and progressively prevalent metabolic risk factors such as hypertension, hyperglycemia, central adiposity, and dyslipidemia (7, 8), the disease burden caused by cardiovascular diseases kept increasing, which has developed into a critical concerning public health issue (9–12). Atherosclerotic cardiovascular disease (ASCVD) includes ischemic heart disease and ischemic stroke (13–15). Because of the same arterial pathological characteristics and risk factors, ASCVD is increasingly regarded as a special type of cardiovascular disease in Chinese and international cardiovascular disease prevention guidelines (16–20). In addition, ASCVD is the pattern that causes the most deaths among all kinds of cardiovascular diseases. In 2016, it caused about 2.4 million deaths in China, which amounts to more than 60% of all cardiovascular disease deaths as well as 25% of all causes of death (21, 22). Therefore, prevention and treatment of ASCVD are the top priorities of management for cardiovascular disease.

Characterized by hypercholesterolemia (high TC), hypertriglyceridemia (high TG), low HDL-C, or high LDL-C, dyslipidemia is a crucial risk factor for ASCVD and one of the three major risk factors that the Healthy China 2030 plan focuses on (23–27), which emphasizes the strategic role of health in China's development and outlines the major principles to achieve this. The disease burden caused by dyslipidemia in China has shown a significant growth trend in recent years (28–30). In 2017, a study showed that 862,759 deaths in China could be attributed to high LDL-C, accounting for 8.25% of all causes of death and 19.71% of cardiovascular disease deaths (31). Economist Intelligence Unit (EIU) report (2018) indicated that CVD had the economic burden of USD 21.7 billion in direct and indirect costs annually in China, of which more than 12% is due to dyslipidemia (32).

Although “co-management of hypertension, diabetes, and hyperlipidemia” was clearly proposed in the Healthy China 2030 plan, the management of dyslipidemia is far from ideal for hypertension and diabetes. The 2017 China Cardiovascular Health Index study showed the prevalence of dyslipidemia (33.7%) exceeded hypertension (26.0%), and diabetes (9.7%), while its awareness rate (14.5%), treatment rate (7.9%), and control rate (5.4%) were all below the corresponding levels of hypertension and diabetes (33). Compared with the standardized management of hypertension and hyperglycemia, that of dyslipidemia is still in a neglected position, and the public's awareness and attention to it need to be strengthened (34–38).

In recent years, researchers have carried out surveys on the epidemiological status of dyslipidemia in China, and many related data have been disclosed. Due to divergent research backgrounds and other reasons, the results varied widely between different studies (39). A comprehensive evaluation on the dyslipidemia epidemiology nationwide in China which may promote our understanding of the epidemiological status of dyslipidemia as well as benefit future research and policy formulation is needed. Consequently, we performed the meta-analyses to comprehensively synthesize the prevalence and management status of dyslipidemia among adults in Chinese Mainland.

## 2. Materials and methods

### 2.1. Search strategy

Based on and Meta-analysis of Observational Studies in Epidemiology (MOOSE) guidelines (40) and the PRISMA statement (41), we conducted the systematic review. Studies on dyslipidemia from six databases were searched, including Web of Science, Embase, PubMed, WanFang, CNKI, and Chinese BioMedical Literature Database. The search strategy was based on a conjunction of “dyslipidemias,” “hyperlipidemias,” “epidemiology,” “prevalence,” “awareness rate,” “treatment rate,” “control rate,” “cross-sectional study,” and so on. The detailed literature retrieval strategy for each database can be found in Supplementary Material. Only studies published in English and Chinese between 1 January 2012 and 31 January 2023 were included.

### 2.2. Inclusion and exclusion criteria

The following information concerning dyslipidemia in Chinese population must be included in eligible studies: prevalence, awareness rate, treatment rate, and control rate. In addition, studies reported that the prevalence of elevated lipoprotein(a) [Lp(a)] was also included.

Eligibility criteria were set as followed: (1) research types: original cross-sectional studies; (2) study participants: Chinese adults; (3) the criteria of prevalence, awareness, treatment, and control rates of dyslipidemia were set based on the latest guideline of Prevention and Treatment for Dyslipidemia in Chinese adults (42).

The exclusion criteria we applied were the following: (1) animal research, (2) non-cross-sectional study, (3) study with a sample size lower than 500, and (4) study on specific occupational groups.

### 2.3. Study identification and data extraction

After combining all searched articles in EndNote *X9* and removing duplicates, two reviewers (QX and YC) independently scanned the titles and abstracts of the retrieved studies for possible eligible studies. Then, the two reviewers independently evaluated the full texts of all potential eligible studies. After discussing and determining the final list of the included studies, QX and YC extract the relevant information by a standardized data collection form, respectively. The extracted information was cross-checked, and a third reviewer (ZY) was responsible for determining any unsettled discrepancies.

The two reviewers (QX and YC) independently extracted the data from studies including but not limited to regular information (such as title, author, and the publication year), characteristics of study (such as study area and number of participants), and characteristics of participants (such as age range, gender, and residential area). Then, the core information was extracted, i.e., the prevalence and rates of awareness, treatment, and control reported in each study. We also extracted these data stratified by age group, sex, region area, and year of screening.

According to the observational study criteria recommended by AHRQ, QX and YC independently evaluated the quality of the included studies (43). With a full score at 11 points, each study was grouped according to its own score. The grouping rules are as follows: good (above 7 points), medium (4–7 points), and poor (below 4 points). In addition, the risk of bias (ROB) of the included studies was evaluated based on the results' quality.

### 2.4. Statistical analysis

We calculated the pooled rates of prevalence, awareness, treatment, and control and corresponding 95% confidence intervals (95% CI) with a systematic analysis approach. Heterogeneity among studies was examined by Cochran's *Q* test and *I^2^* statistic. The *I^2^* statistic value at 25%, 50%, and 75% indicated a low, moderate, and high degree of heterogeneity, respectively (44, 45). The random-effect model will be used if the result suggested a high degree of heterogeneity (*I^2^* > 50%). Otherwise, the fixed-effect model will be used (46).

To address heterogeneity between studies, subgroup analyses by age group, sex, geographic region, and the year of screening were performed. Afterward, a meta-regression was conducted, and the added variables include sex ratio (males vs. females), the year of screening, geographic area (southern vs. northern China), studies' quality score, and sample size. Finally, we performed a sensitivity analysis by evaluating the influence of the study's quality on the pooled estimates. Funnel plots and *Egge*r's test were used to assess the risk of publication bias. We set the significance level at a *P* < 0.05. *Stata* and *SPSS* software were used to perform statistical analyses.

## 3. Results

### 3.1. Characteristics of included studies

#### 3.1.1. Search results

**Figure 1:**
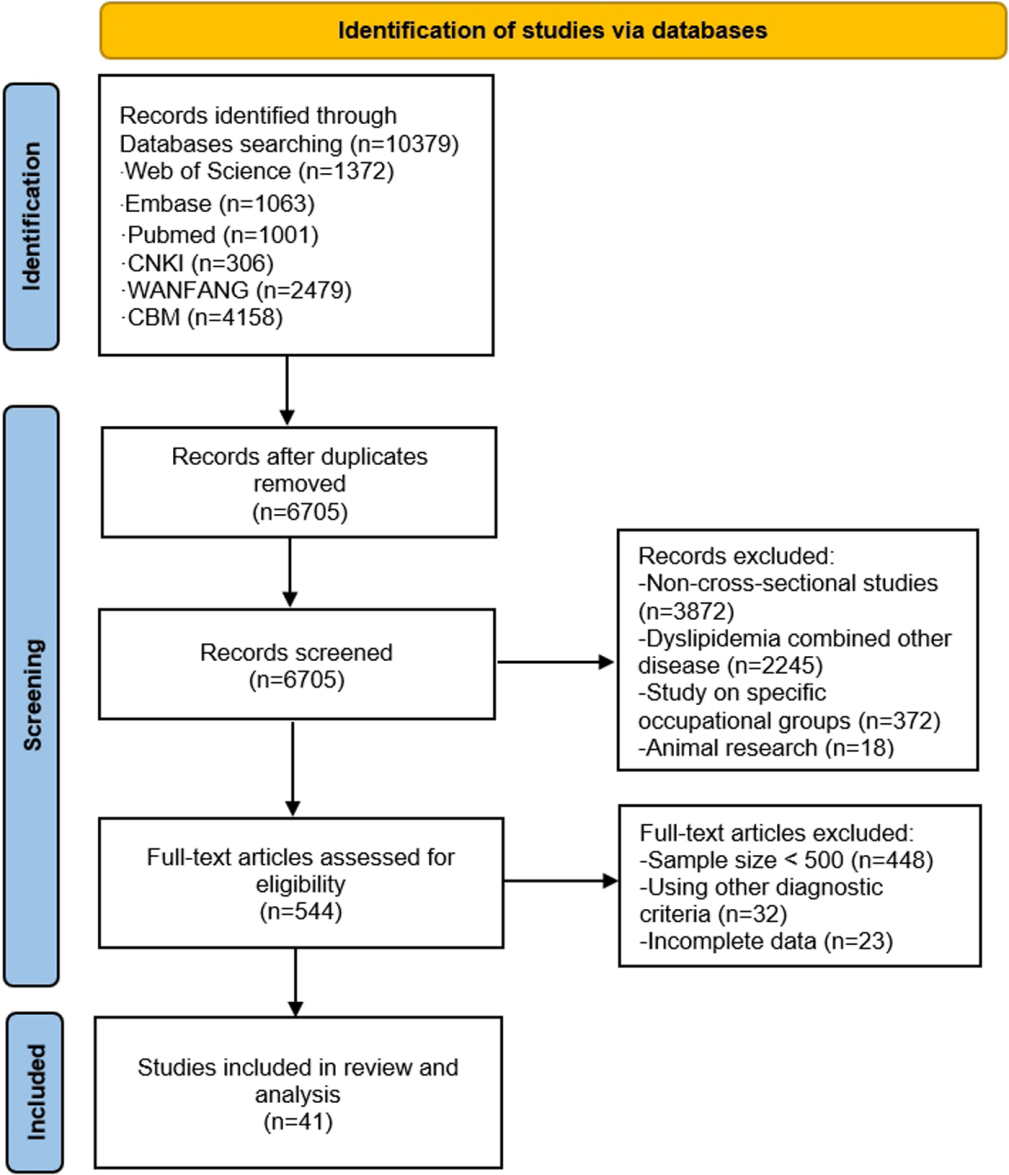
PRISMA flow chart of included studies.

A total of 10,379 studies from all databases were searched and gathered in EndNote *X9* software. Initially, 3,674 duplicates were removed, and then 6,161 studies were eliminated after reading the titles and abstracts, leaving 544 possibly qualified articles. Studies using other diagnostic criteria, reporting incomplete data or with a sample size of <500, were further excluded after referring to the full text. Finally, 41 studies (47–87) were included for analysis which involved a total of 1,310,402 Chinese adults. The search selection process is displayed in Figure 1.

#### 3.1.2. Studies characteristics

Table 1 represented the characteristics of all included studies. Among all included 41 studies, 30 were published in Chinese (47–60, 63, 64, 68–70, 73–79, 81, 85–87), and 11 were in English (61, 62, 65–67, 71, 72, 80, 82–84). All studies were published between 2012 and 2023. As for study area, 14 studies (47, 49, 50, 58, 61, 63, 69, 70, 72, 75–77, 83, 85) were conducted on the populations of northern China, while 17 studies (48, 51–57, 59, 68, 71, 73, 74, 78, 84, 86, 87) focused on southern counterparts, and 10 nationwide studies (60, 62, 64–67, 79–81, 82) were conducted.

**Table 1:** Characteristic of 41 included studies of the epidemiology of dyslipidemia in Chinese adults.

No.	References	Publication year	Screening year	Region	Area	Age range	Case	Sample size
1	Liu R et al.	2021	2018	Shaanxi	Northern	≥18 years	1915	6,040
2	Luo SY et al.	2014	2010	Guangxi	Southern	≥18 years	1,907	3,599
3	Zhang R et al.	2018	2013–2014	Xinjiang	Northern	≥18 years	1,854	4,120
4	Zhang GH et al.	2017	2013	Shandong	Northern	≥18 years	3,535	11,223
5	Xu W et al.	2020	2015	Anhui	Southern	≥18 years	2,258	7,404
6	Li WY et al.	2015	2010–2011	Fujian	Southern	≥18 years	3,694	6,016
7	Mo JF et al.	2013	2010	Guangdong	Southern	≥18 years	2,171	3,577
8	Liu T et al.	2017	2011	Guizhou	Southern	≥18 years	5,392	9,280
9	Pan JJ et al.	2017	2013	Hubei	Southern	≥18 years	1,938	5,926
10	Wang YY et al.	2019	2014	Jiangsu	Southern	≥18 years	3,170	8,299
11	Chen YY et al.	2013	2010	Jiangxi	Southern	≥18 years	1,821	3,000
12	Yang XY et al.	2016	2012	Tianjin	Northern	≥18 years	2,592	8,968
13	Zhang XW et al.	2012	2010	Zhejiang	Southern	≥18 years	8,701	17,437
14	Dai Z et al.	2018	2011	–	National	≥18 years	3,459	8,669
15	Pan JH et al.	2018	2013	Shanxi	Northern	≥18 years	1,749	4,105
16	Pan L et al.	2016	2010	–	National	≥18 years	15,786	43,368
17	Lai YX et al.	2012	2007	Liaoning	Northern	≥20 years	1,542	2,989
18	Li SN et al.	2019	2012–2015	–	National	≥35 years	10,298	29,678
19	Sampson Opoku et al.	2019	2014	–	National	≥40 years	59,160	1,36,945
20	Xing LY et al.	2020	2017–2019	–	National	≥40 years	6,729	18,796
21	Song PG et al.	2019	2011	–	National	≥45 years	4,077	9,525
22	Long XT et al.	2022	2019–2020	Yunnan	Southern	≥60 years	3,282	9,709
23	Zhao Y et al.	2017	2014	Beijing	Northern	18–65 years	8220	18,809
24	Sun WF et al.	2016	2013–2014	Gansu	Northern	20–74 years	11,907	31,417
25	Huang C et al.	2021	2013–2014	Sichuan and Chongqing	Southern	35–79 years	2,801	10,221
26	Zhang J et al.	2020	2010	Zhejiang	Southern	≥18 years	–	17,437
27	Li JB et al.	2022	2018	Henan	Northern	≥18 years	–	6,809
28	Li JH et al.	2012	2010	–	National	≥18 years	–	51,818
29	Ma JJ et al.	2016	2012–2014	Xinjiang	Northern	≥35 years	–	4,314
30	Zhang M et al.	2016	2014	Guizhou	Southern	≥40 years	–	5,126
31	Sampson Opoku et al.	2021	2015	–	National	≥40 years	–	135,403
32	Xie J et al.	2017	2014	Beijing	Northern	18–65 years	–	18,809
33	He H et al.	2014	2012	Jilin	Northern	18–79 years	–	7,319
34	Zhang YF et al.	2021	2017–2020	Fujian	Southern	35–75 years	–	119,638
35	Lu Y et al.	2018	2011–2012	–	National	45–75 years	–	12,654
36	Lin LJ et al.	2023	2010–2017	–	National	≥18 years	–	411,643
37	Guo CY et al.	2021	2017	Beijing and Tianjin and Hebei	Northern	≥18 years	–	25,343
38	Xuan LP et al.	2020	2010	Shanghai	Southern	≥40 years	–	6,257
39	Niu DR et al.	2018	2015	Shandong	Northern	<100 years	–	63,882
40	Li YY et al.	2014	2012–2013	Fujian	Southern	<100 years	–	3,944
41	Chen JW et al.	2019	2018–2019	Guangzhou	Southern	≥25 years	–	886

**Figure 2:**
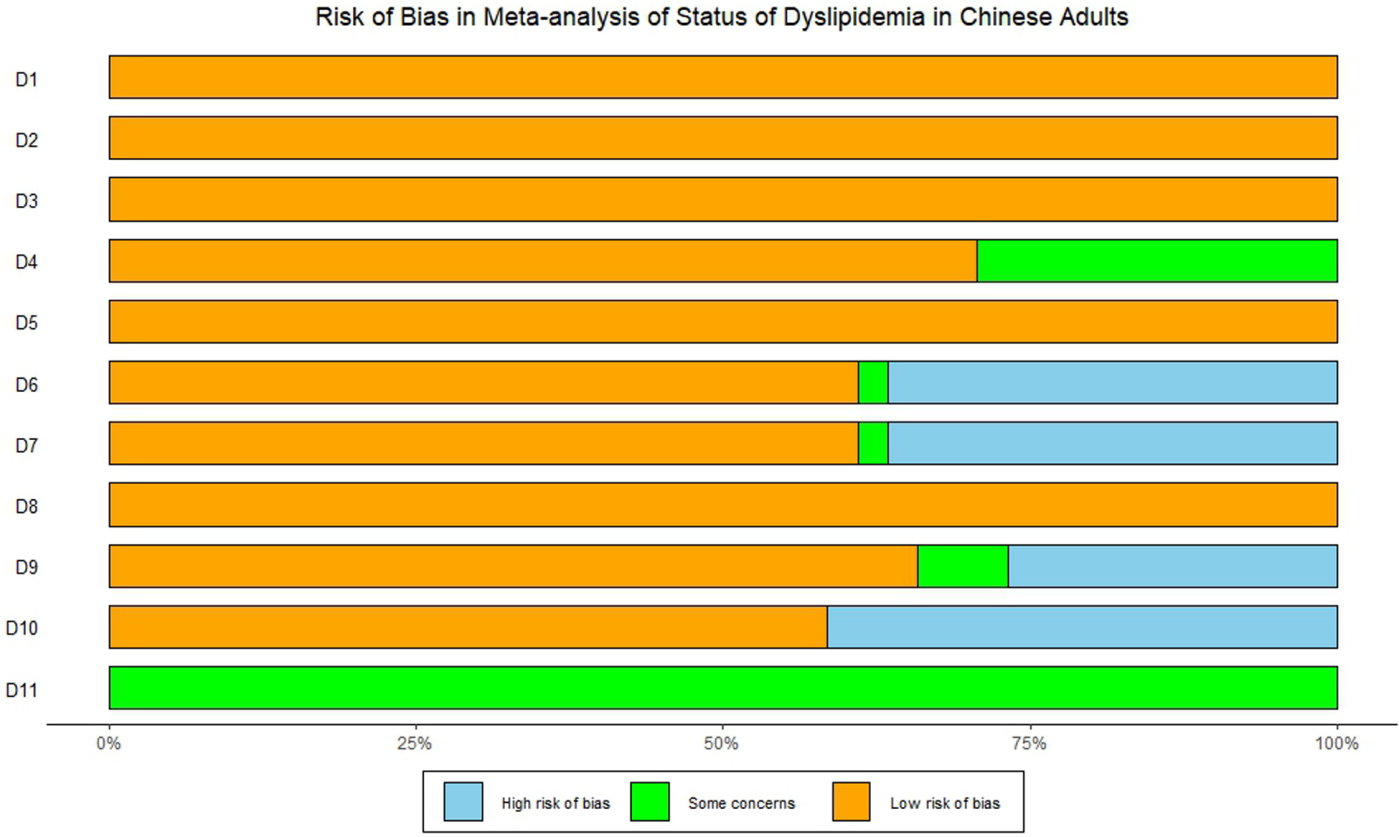
Summary plot of risk bias.

The results of quality evaluation of the included studies were displayed in Table 2. A total of 29 studies scored above 7 and were consequently rated as high-quality, while 12 studies were rated as medium-quality, and no studies of low-quality were observed. The studies' average quality score was 8.07, while the standard deviation was 1.09. Since no study was evaluated as low-quality, all studies were included for further analyses. Figure 2 showed the summary plots of assessment for risk bias.

**Table 2:** Quality evaluation results of systematic review of epidemiology of dyslipidemia in Chinese adults.

Study ID	References	D1	D2	D3	D4	D5	D6	D7	D8	D9	D10	D11	Overall
1	Liu R et al.	1	1	1	1	1	1	0	1	0	0	Unclear	7
2	Luo SY et al.	1	1	1	1	1	0	1	1	1	1	Unclear	9
3	Zhang R et al.	1	1	1	Unclear	1	0	1	1	1	1	Unclear	8
4	Zhang GH et al.	1	1	1	1	1	0	0	1	1	0	Unclear	7
5	Xu W et al.	1	1	1	1	1	1	1	1	0	0	Unclear	8
6	Li WY et al.	1	1	1	Unclear	1	1	0	1	0	0	Unclear	6
7	Mo JF et al.	1	1	1	1	1	1	1	1	0	1	Unclear	9
8	Liu T et al.	1	1	1	Unclear	1	0	0	1	1	1	Unclear	7
9	Pan JJ et al.	1	1	1	Unclear	1	0	0	1	1	1	Unclear	7
10	Wang YY et al.	1	1	1	1	1	0	1	1	1	1	Unclear	9
11	Chen YY et al.	1	1	1	Unclear	1	1	0	1	0	0	Unclear	6
12	Yang XY et al.	1	1	1	1	1	0	1	1	1	1	Unclear	9
13	Zhang XW et al.	1	1	1	1	1	1	1	1	1	1	Unclear	10
14	Dai Z et al.	1	1	1	1	1	1	0	1	1	0	Unclear	8
15	Pan JH et al.	1	1	1	Unclear	1	0	1	1	1	0	Unclear	7
16	Pan L et al.	1	1	1	1	1	0	0	1	1	1	Unclear	8
17	Lai YX et al.	1	1	1	1	1	0	1	1	0	0	Unclear	7
18	Li SN et al.	1	1	1	Unclear	1	1	1	1	1	0	Unclear	8
19	Sampson Opoku et al.	1	1	1	1	1	1	1	1	1	0	Unclear	9
20	Xing LY et al.	1	1	1	1	1	0	1	1	0	1	Unclear	8
21	Song PG et al.	1	1	1	1	1	0	1	1	1	1	Unclear	9
22	Long XT et al.	1	1	1	Unclear	1	1	0	1	1	0	Unclear	7
23	Zhao Y et al.	1	1	1	1	1	0	1	1	1	0	Unclear	8
24	Sun WF et al.	1	1	1	1	1	1	0	1	1	1	Unclear	9
25	Huang C et al.	1	1	1	Unclear	1	0	0	1	1	0	Unclear	6
26	Zhang J et al.	1	1	1	1	1	1	0	1	0	1	Unclear	8
27	Li JB et al.	1	1	1	1	1	1	0	1	1	1	Unclear	9
28	Li JH et al.	1	1	1	1	1	1	0	1	0	0	Unclear	7
29	Ma JJ et al.	1	1	1	1	1	1	1	1	1	0	Unclear	9
30	Zhang M et al.	1	1	1	Unclear	1	1	1	1	0	1	Unclear	8
31	Sampson Opoku et al.	1	1	1	1	1	1	1	1	1	0	Unclear	9
32	Xie J et al.	1	1	1	Unclear	1	1	1	1	1	0	Unclear	8
33	He H et al.	1	1	1	1	1	1	1	1	1	0	Unclear	9
34	Zhang YF et al.	1	1	1	Unclear	1	1	0	1	0	0	Unclear	6
35	Lu Y et al.	1	1	1	1	1	0	1	1	1	0	Unclear	8
36	Lin LJ et al.	1	1	1	1	1	1	1	1	1	1	Unclear	10
37	Guo CY et al.	1	1	1	1	1	1	1	1	1	0	Unclear	9
38	Xuan LP et al.	1	1	1	1	1	1	1	1	1	1	Unclear	10
39	Niu DR et al.	1	1	1	1	1	1	Unclear	1	Unclear	1	Unclear	8
40	Li YY et al.	1	1	1	1	1	1	1	1	Unclear	1	Unclear	9
41	Chen JW et al.	1	1	1	1	1	Unclear	1	1	Unclear	1	Unclear	8

***D1:*** Define the source of information (survey, record review). ***D2:*** List inclusion and exclusion criteria for exposed and unexposed subjects (cases and controls) or refer to previous publications. ***D3:*** Indicate time period used for identifying patients. ***D4:*** Indicate whether or not subjects were consecutive if not population-based. ***D5:*** Indicate if evaluators of subjective components of study were masked to other aspects of the status of the participants. ***D6:*** Describe any assessments undertaken for quality assurance purposes (e.g., test/retest of primary outcome measurements). ***D7:*** Explain any patient exclusion from analysis. ***D8:*** Describe how confounding was assessed and/or controlled. ***D9:*** If applicable, explain how missing data were handled in the analysis. ***D10:*** Summarize patient response rates and completeness of data collection. ***D11:*** Clarify what follow-up, if any, was expected and the percentage of patients for which incomplete data or follow-up was obtained.

### 3.2. Prevalence of dyslipidemia

**Figure 3:**
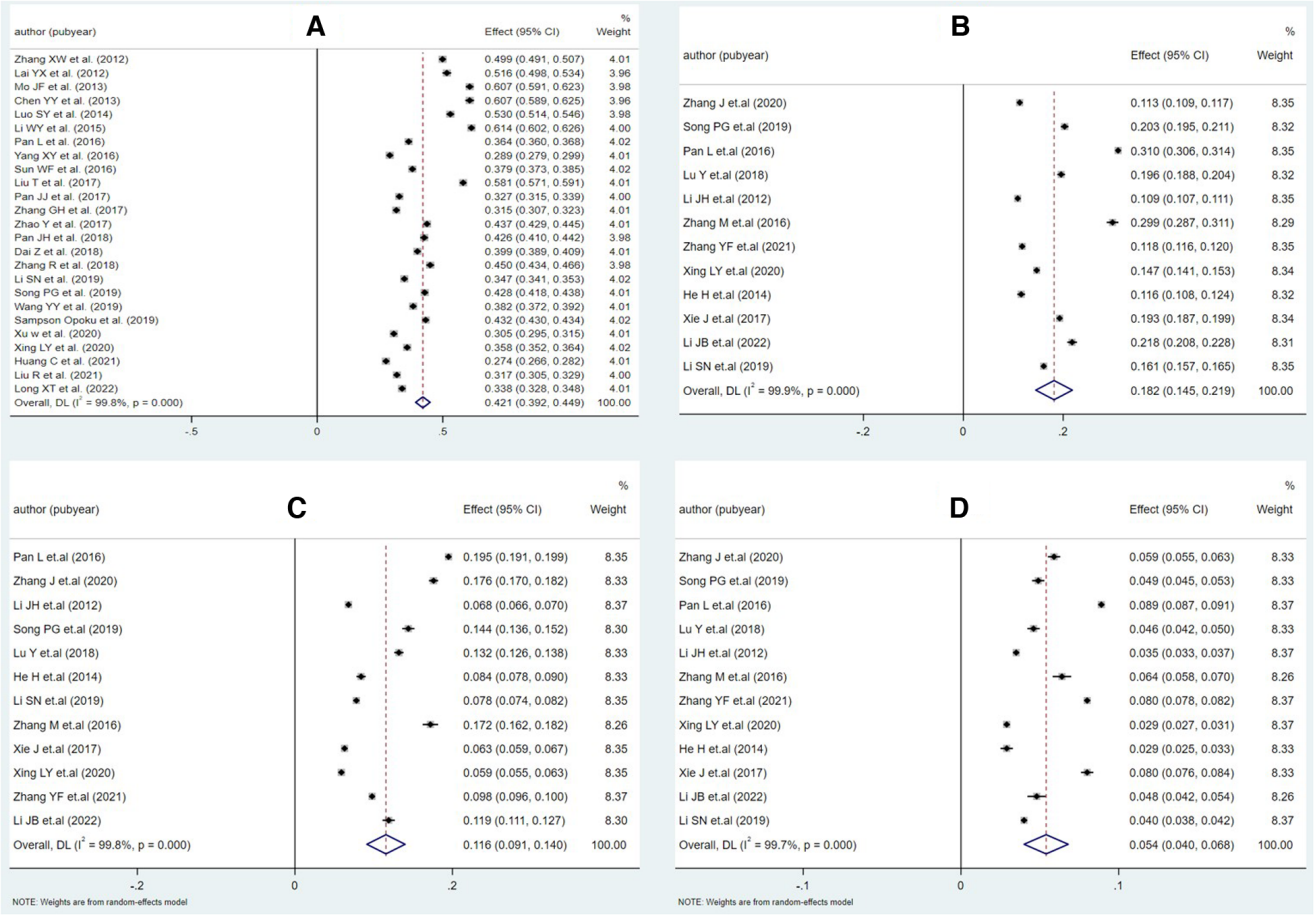
Forest plot of meta-analysis for each part of epidemiology of dyslipidemia in Chinese adults. (**A**) Prevalence, (**B**) awareness rate, (**C**) treatment rate, and (**D**) control rate.

**Figure 4:**
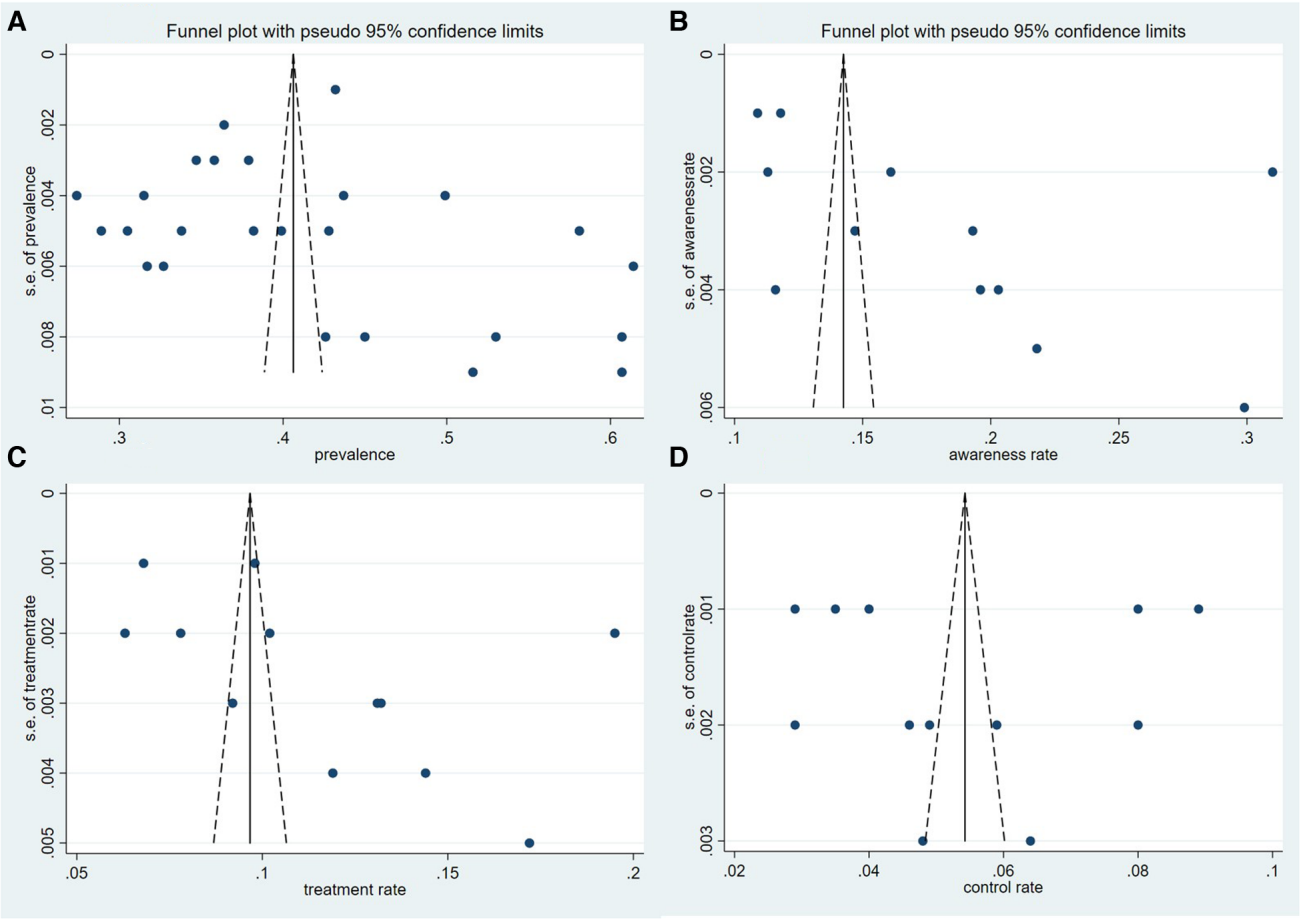
Funnel plot of each theme. (**A**) Prevalence, (**B**) awareness rate, (**C**) treatment rate, and (**D**) control rate.

Twenty-five articles detailed the dyslipidemia prevalence of their population (see Table 3). The meta-analysis suggested that the pooled prevalence of dyslipidemia among adults in Chinese Mainland was 42.1% (95% CI: 39.2%– 44.9%), while the heterogeneity between studies was extremely high (*I^2^* = 99.8%, *P* < 0.001). The forest plots of the pooled prevalence and rates of awareness, treatment, and control were displayed in Figure 3, and the corresponding funnel plots were shown in Figure 4. The *Egger*'s test and funnel plots (Figure 3A) demonstrated that no significant publication bias on the prevalence of dyslipidemia was found (*P* = 0.996).

**Table 3:** Prevalence of dyslipidemia according to different categories.

Category	Subgroup	Number of studies	Prevalence (95% CI) (%)	Sample	*I^2^* (*%*)	*P*
Total		25	42.1 (39.2–44.9)	419,120	99.8	
Sex	Male	24	47.3 (44.0–50.6)	194,757	99.5	<0.001
	Female	24	38.8 (34.3–43.3)	214,121	99.8	
Age-specific group (y)	18–44	10	40.1 (33.1–47.1)	39,388	99.5	<0.001
	45–59	10	45.2 (39.7–50.7)	33,466	99.1	
	≥60	10	44.9 (38.8–50.9)	19,658	98.7	
Geographic region	Northern	8	39.1 (34.5–43.7)	87,671	99.4	<0.001
	Southern	11	46.0 (38.2–53.9)	84,468	99.8	
	Urban	21	46.3 (43.2–49.5)	167,241	99.3	<0.001
	Rural	21	41.3 (37.3–45.2)	218,097	99.7	
Screening year	2007–2010	7	53.4 (44.2–62.6)	79,986	99.8	<0.001
	2011–2013	8	38.9 (32.7–45.1)	87,374	99.7	
	2014–2020	10	36.7 (33.0–40.5)	251,760	99.7	
Types	Hypercholesterolemia (TC)	23	8.3 (6.9–9.6)	393,384	99.6	<0.001
	Hypertriglyceridemia (TG)	23	15.4 (12.5–18.3)	393,384	99.8	
	Low levels of high-density lipoprotein cholesterol (HDL-C)	23	24.5 (21.0–28.1)	393,384	99.9	
	High levels of low-density lipoprotein cholesterol (LDL-C)	23	7.1 (5.9–8.2)	393,384	99.5	
	Elevated Lp(a)	6	19.4 (16.8–22.1)	511,955	99.5	

*P*, *P*-value of *z*-test.

Table 3 detailed the results of subgroup analysis stratified by gender, age group, geographic area, year of screening, and types of dyslipidemia. Males had higher pooled prevalence (47.3%, 95% CI: 44.0%–50.6%) of dyslipidemia than females (38.8%, 95% CI: 34.3%–43.3%), and the difference was statistically significant (*P* < 0.001). The pooled prevalence for specific age ranges was 45.2% (95% CI: 39.7%–50.7%) for subjects aged 45–59 years, which was the highest and similar to that of subjects aged ≥60 years (44.9%, 95% CI: 38.8%–50.9%), and subjects aged 18–44 years had the lowest pooled prevalence (40.1%, 95% CI: 33.1%–47.1%). Populations living in the southern area of China had higher pooled prevalence of dyslipidemia (46.0%, 95% CI: 38.2%–53.9%) than those living in northern China (39.1%, 95% CI: 34.5%–43.7%). In addition, the pooled prevalence of urban residents (46.3%, 95% CI: 43.2%–49.5%) was high than that of rural residents (41.3%, 95% CI: 37.3%–45.2%). In addition, the pooled prevalence of dyslipidemia decreased with time, which was 53.4% (95% CI: 44.2%–62.6%) during 2007–2010, decreasing to 38.9% (95% CI: 32.7%–45.1%) during 2011–2013, and decreasing further to 36.7% (95% CI: 33.0%–40.5%) during 2014–2020.

**Figure 5:**
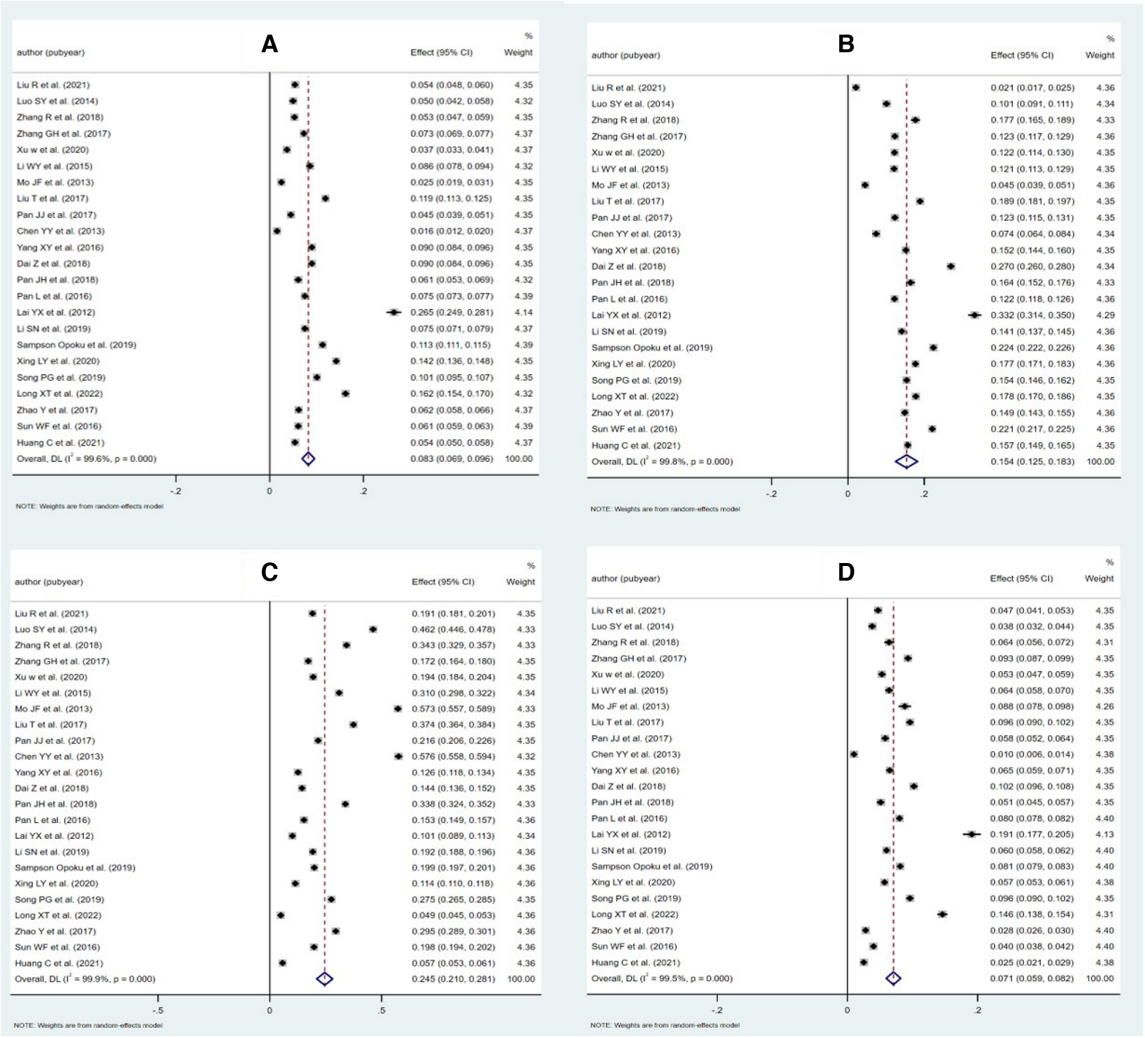
Forest plots for the pooled prevalence of different types of dyslipidemia. (**A**) Hypercholesterolemia (TC), (**B**) hypertriglyceridemia (TG), (**C**) low levels of high-density lipoprotein cholesterol (HDL-C), and (**D**) high levels of low-density lipoprotein cholesterol (LDL-C).

**Figure 6:**
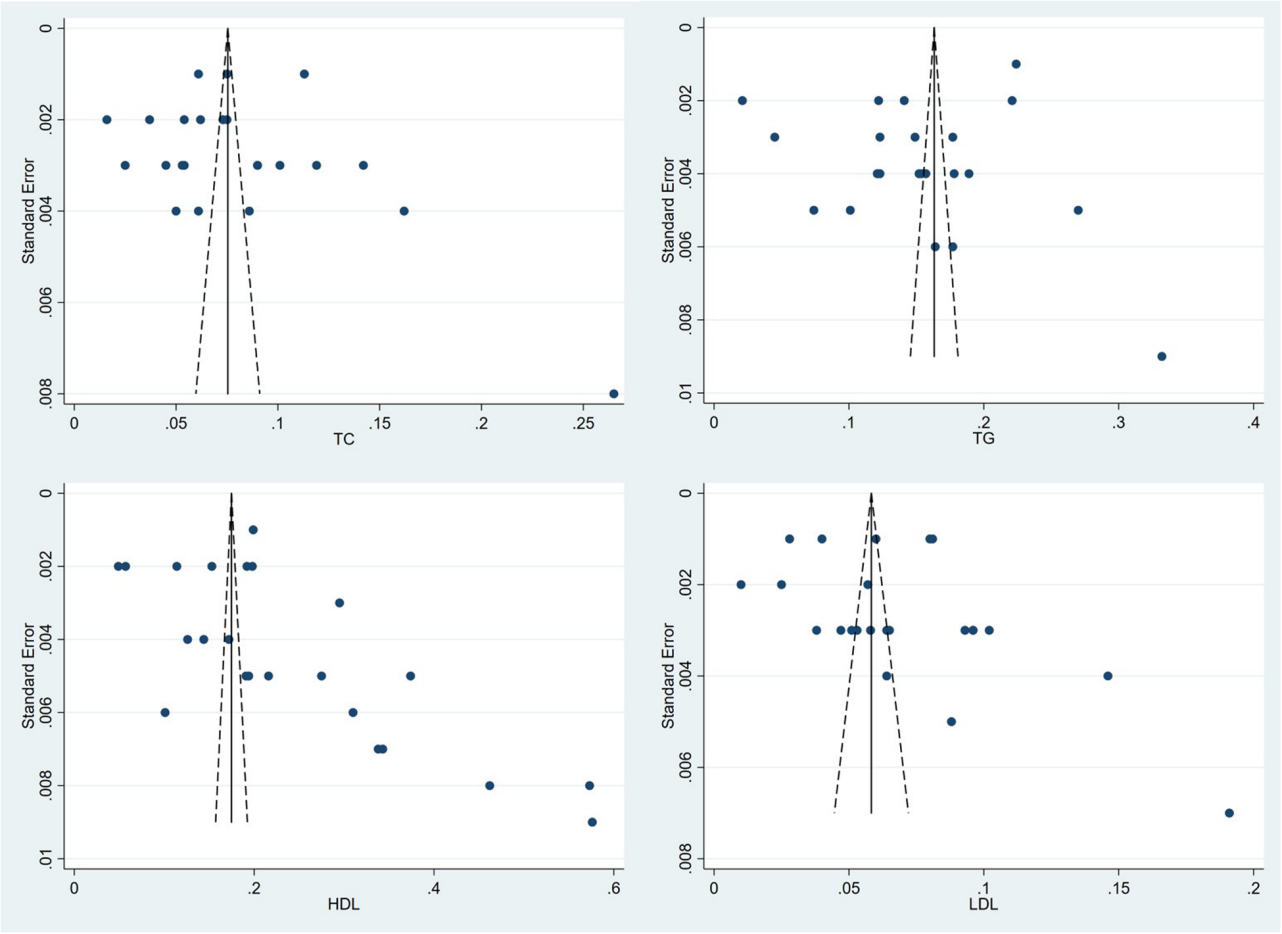
Funnel plots for the pooled prevalence of different types of dyslipidemia.

The pooled prevalence of different types of dyslipidemia varied widely. HDL-C was the highest at 24.5% (95% CI: 21.0%–28.1%), followed by hypertriglyceridemia at 15.4% (95% CI: 12.5%–18.3%), and the pooled prevalence of hypercholesterolemia and LDL-C were lower at 8.3% (95% CI: 6.9%–9.6%) and 7.1% (95% CI: 5.9%–8.2%), respectively. The forest plots for the pooled prevalence of different types of dyslipidemia were displayed in Figure 5. Corresponding funnel plots were shown in Figure 6. We also calculated the pooled prevalence of elevated Lp(a), which was defined as a serum Lp(a) value of >30 mg/dl according to the latest guideline of Prevention and Treatment for Dyslipidemia in Chinese adults (42). The results showed a pooled prevalence between that of HDL-C and hypertriglyceridemia at 19.4% (95% CI: 16.8%–22.1%). Forest plot and funnel plot for the prevalence of elevated Lp(a) could be searched in Supplementary Material.

### 3.3. Dyslipidemia awareness, treatment, and control

According to the related information released in 12 surveys, we arrived at pooled rates of awareness, treatment, and control of dyslipidemia at 18.2%, 11.6%, and 5.4%, respectively (see Table 4). The funnel plots (see Figure 4) and the *Egger*'s tests demonstrated that no significant publication bias on the rates was observed (*P* = 0.072, 0.110 and 0.958, respectively).

**Table 4:** Awareness, treatment, and control rates of dyslipidemia according to different categories.

Category	Subgroup	Number of studies	Prevalence (95% CI) (%)	Sample	*I^2^* (*%*)	*P*
Awareness rate						
Sex	Male	10	15.7 (12.0–19.5)	149,684	99.8	<0.001
	Female	10	18.5 (14.5–22.5)	173,513	99.8	
Geographic region	Urban	8	19.1 (16.3–21.9)	94,429	99.1	<0.001
	Rural	8	11.1 (9.2–13.0)	166,591	99.1	
	Northern	4	16.8 (12.8–20.8)	51,733	99.2	0.055
	Southern	3	17.6 (12.5–22.7)	142,201	99.8	
Screening year	2009–2013	6	17.4 (10.0–24.9)	134,802	99.9	<0.001
	2014–2020	6	18.9 (15.1–22.7)	210,489	99.7	
Total		12	18.2 (14.5–21.9)	340,977	99.9	
Treatment rate						
Sex	Male	10	9.7 (7.5–12.0)	149,684	99.6	<0.001
	Female	10	12.3 (9.7–15.0)	173,513	99.9	
Geographic region	Urban	8	12.1 (6.8–17.4)	94,429	99.9	<0.001
	Rural	8	7.7 (6.4–9.1)	166,591	100.0	
	Northern	4	9.4 (7.0–11.7)	51,733	99.2	<0.001
	Southern	3	13.3 (9.6–17.1)	142,201	99.3	
Screening year	2009–2013	6	12.7 (7.8–17.6)	134,802	99.9	<0.001
	2014–2020	6	10.5 (8.7–12.3)	210,489	99.2	
Total		12	11.6 (9.1–14.0)	340,977	99.8	
Control rate						
Sex	Male	10	4.2 (3.0–5.5)	149,684	99.2	<0.001
	Female	10	6.4 (4.5–8.3)	173,513	99.7	
Geographic region	Urban	8	5.5 (3.1–8.0)	94,429	99.5	<0.001
	Rural	8	3.6 (2.5–4.7)	166,591	99.1	
	Northern	4	4.6 (2.2–7.1)	51,733	99.5	<0.001
	Southern	3	6.8 (5.3–8.3)	142,201	98.0	
Screening year	2009–2013	6	5.1 (2.9–7.4)	134,802	99.7	0.001
	2014–2020	6	5.7 (3.6–7.7)	210,489	99.7	
Total		12	5.4 (4.0–6.8)	340,977	99.7	

*P*, *P*-value of *z*-test.

The pooled rates of awareness, treatment, and control in females (18.5%, 12.3%, and 6.4%, respectively) were higher than those in males (15.7%, 9.7%, and 4.2%, respectively). Urban residents had higher pooled rates of awareness, treatment, and control than the rural residents (*P* < 0.001). Southern residents had higher pooled rates of treatment and control (13.3% and 6.8%, respectively) than the northern populations (9.4% and 4.6%, respectively), while the difference of awareness rates was not statistically significant among them (*P *= 0.055). The pooled rates of awareness and control increased with time. The pooled rates are 17.4% and 5.1%, respectively, during 2009–2013, increasing to 18.9% and 5.7% during 2014–2020. However, the pooled rate of treatment dropped from 12.7% to 10.5% over the same period.

### 3.4. Sensitivity analysis and meta-regression

In the sensitivity analysis, we excluded four citations with a quality score of 6 which was the lowest among all studies. After omitting these studies, we discovered a slight decrease in the pooled prevalence (from 42.1% to 41.0%, *P* = 0.653). Funnel plot combined with *Egger*'s test (*P* = 0.639) indicated that no significant publication bias was noted. The pooled prevalence of hypercholesterolemia, hypertriglyceridemia, HDL-C, and LDL-C changed to 8.7%, 15.9%, 23.5%, and 7.6% from 8.3%, 15.4%, 24.5%, and 7.1%, respectively. The pooled rates of awareness, treatment, and control also changed slightly (from 18.2%, 11.6%, and 5.4% to 18.8%, 11.8%, and 5.2%, respectively) after omitting these studies. The results indicated that the pooled prevalence and rates of awareness, treatment, and control had good stability.

Finally, a meta-regression was performed to address the high level of heterogeneity between studies (*I^2^* = 98.7%–100.0%). Five variables (sex ratio, the year of screening, geographic area, studies' quality score, and sample size) were included in the analyses. The results of meta-regression demonstrated that only the year of screening variable had a significant impact on the heterogeneity (*P* = 0.011) (see Table 5).

**Table 5:** Results of meta-regression for the prevalence of dyslipidemia.

Covariate	Meta-regression coefficient	95% confidence interval	*P*
Year of screening	−0.086	−0.147 to −0.024	0.011
Sex ratio (male vs. female)	−0.110	−0.353 to 0.134	0.346
Area (southern vs. northern)	−0.034	−0.138 to 0.070	0.490
Sample size, continuous	8.16e^−7^	−6.63e^−6^ to 8.26e^−6^	0.815
Quality score	9.56e^−5^	−0.056 to 0.056	0.997

*P*, *P*-value of meta-regression.

## 4. Discussion

For the first time, this study comprehensively summarized and analyzed the epidemiological studies on dyslipidemia in recent years and explored its potential influencing factors. With a total number of 1,310,402 participants from different cross-sectional studies, our systematic review incorporated 41 studies conducted in multiple provinces of Chinese Mainland over the past decade. The results of the meta-analyses suggested a high-degree of prevalence of dyslipidemia in Chinese adults along with unacceptably low rates of awareness, treatment, and control. Results from this study would be a timely alarm since a comprehensive evaluation on the dyslipidemia epidemiology nationwide in China which may promote our understanding of its epidemiological status and benefit future research and policy formulation is still lacking. In 2014, the pooled prevalence of dyslipidemia among adults in Chinese Mainland is 42.1%, which is very close to the research results of Huang et al. (41.9%) (88). The deepening of aging population degree and the great changes in residents' living habits (including but not limited to diet and physical activity) could lead to the highly prevalent dyslipidemia in recent years (89–94). By comparison with other developed countries, the dyslipidemia prevalence in Chinese adults was still lower than that reported in the United States (54.9%) (95) but much higher than that reported in Korea (16.6%) (96) and Japan (27.1%) (97). Among all types of dyslipidemia, HDL-C was the most prevalent, with a pooled estimate of 24.5%, followed by hypertriglyceridemia (15.4%), hypercholesterolemia (8.3%), and LDL-C (7.1%). In accordance with the results from surveys of Chinese Mainland in 2008–2019 (65, 98, 99), the more prevalent types of dyslipidemia in China were still HDL-C and hypertriglyceridemia. As the risk of ASCVD will be increased by all types of dyslipidemia, it is essential to treat them with applicable interventions, both clinical and non-clinical (25, 88).

The pooled estimates of dyslipidemia prevalence between different genders, age groups, and regions were calculated and analyzed. We found that males had higher pooled prevalence than females (47.3% vs. 38.8%; *P* < 0.001), which is consistent with the previous studies' results (100–103). Many factors can attribute to such difference, for instance, males are more likely to possess unhealthy behaviors than females, including but not limited to lack of vegetables and fruits in diet and fewer physical activity. Such factors could contribute to higher prevalence of many metabolic diseases in males than females, including dyslipidemia (104–108). Researches showed that estrogen changes the vascular permeability by increasing nitrous oxide production which retains a healthful lipoprotein profile (109, 110). Nonetheless, such protective mechanisms will disappear after menopause, resulting in an ascending risk for suffering cardiovascular diseases. For example, the levels of TC, TG, LDL­C, and VLDL-C aming post­menopausal women appear an upward trend, while that of HDL-C significantly decreases. Consequently, the prevalence of dyslipidemia in women often showed a dramatic increase after menopause, even surpassing that of men of the same age (60, 62, 65, 99). The systematic review revealed that southern and urban residents were more likely to suffer from dyslipidemia than their counterparts, which was a result in line with previous findings (111, 112). Another study in China suggested that economically developed areas (e.g., southeast area of China) tend to possess higher burden of dietary chronic conditions, such as dyslipidemia and obesity (113). In addition, healthcare facilities are more accessible for residents in highly urbanized and economically vibrant cities, resulting in more diagnosis and hence higher reporting rate of dyslipidemia in urban and southern areas. It is worth noting that in recent years, dyslipidemia has become more and more prevailing in Chinese youngsters. A cross-sectional study conducted in Wenzhou, Zhejiang Province, showed a prevalence at 34.11% among 7,859 young adults (114), which was much higher than the figure in Mainland China 10 years before that, as well as other Asian developing countries (115, 116). In Beijing, a study of 3,249 children aged 6–18 years showed that the prevalence of dyslipidemia was 28.9%, higher than 18.8% in 2004 (117). The younger trend of dyslipidemia may be partly attributed to the westernization of young people's lifestyle, such as dietary patterns (114). It is indisputable that corresponding risk factor intervention project should be developed based on the characteristics of young people and further screening and management programs should be strengthened.

One of the key findings of this study is a summary estimation of the pooled prevalence of elevated Lp(a) in Chinese adults, although it is not included in the definition of dyslipidemia. According to the latest guideline of Prevention and Treatment for Dyslipidemia in Chinese adults (42), we set the cutoff value at >30 mg/dl and got a pooled prevalence at 19.4%, which was much lower than that in the United States (35%) (118). As a novel lipid biomarker which could promote the formation of atherosclerosis and thrombosis, Lp(a) was considered the core pathogenesis of ASCVD and deemed as a reversible risk factor (119). Based on seven randomized controlled trials and 29,069 patients taking statin medication, one meta-analysis found that elevated Lp(a) can still increase the risk of CVD, despite a controlled LDL-C level (120). Due to the fact that the concentration level of Lp (a) in plasma is mainly caused by genetic factors, it is relatively stable throughout life (121). Chinese researchers recommended that people consider at least once measurement of Lp(a) to identify individuals who have inherited extremely increased levels of Lp(a) (≥180 mg/dl), which may bring an extremely high lifetime risk of ASCVD (119).

Publicizing the harm of dyslipidemia to the general public along with encouraging a healthier lifestyle is the basic strategy to prevent dyslipidemia and ASCVD. For patients with dyslipidemia, the focus is to control the blood lipid level to the normal range through therapeutic approaches, such as taking statins. Therefore, improving residents' awareness, treatment, and control of dyslipidemia is the key to effective management of dyslipidemia. The pooled rates of awareness, treatment, and control of dyslipidemia among Chinese adults are 18.2%, 11.6%, and 5.4%, respectively, which are slightly higher than the research results of Zhao et al. (10.9%, 6.8%, and 3.5%, respectively) in Chinese Mainland in 2010 but much lower than that reported in the United States (73.3%, 54.1%, and 35.7%) and also lower than the results from Argentina (37.3%, 36.6%, and 20.0%) and South Korea (29.4%, 17.0%, and 13.9%, respectively) (79, 122, 123). In accordance with previous studies (80, 88), our study suggested that women had higher awareness rates of dyslipidemia than men and tend to receive corresponding treatment, which further contributed to better control of their condition. Various factors can account for such phenomenon, for instance, studies demonstrated that women more frequently seek medical services than men (124). In addition, women are more likely to have a health insurance as well as an ongoing source for primary care than men (125). Therefore, we suggested strengthening the promotion and management of dyslipidemia among male residents in China. In line with the previous studies (62, 80), our results showed that urban residents had significantly higher rates of awareness, treatment, and control than rural residents. Similarly, such urban–rural difference was found in a couple of findings from other countries (119, 126, 127). The differences in rates between urban and rural areas may be partly attributed to the gap between residents' economy and education level (128). A prior study suggested a gap in statin availability between urban and rural areas, which may support evidence that rural residents generally have difficulty accessing health care, especially in developing countries (129, 130). Therefore, it is suggested to attach more importance to the dyslipidemia management in rural areas, such as increasing the availability of drugs. The significance of this study has been highlighted by the extremely low rates of awareness, treatment, and control discovered, which may imply some disparities and deficiencies in dyslipidemia management in China. The results of a study on 99,655 patients with dyslipidemia who had prescribed statins in Tianjin showed that although high adherence to medications can reduce the risk of major adverse cardiovascular events, only 5.4% of the patients insist on taking statins for more than 50% of the days (131). Results of China Dyslipidemia Survey (DYSIS) signified that among the outpatients in China who had regularly taken lipid-lowering drugs, statin monotherapy accounted for 97.96%, while combination therapy accounted for only 2.04%. In addition, despite the steady lipid-lowering therapy, the vast majority of patients still had at least one manifestation of dyslipidemia (132). China PEACE Million Persons Project investigated the accessibility of lipid-lowering drugs in primary healthcare institutions of China. Only 49.7% of total number of 3,041 primary care institutions stocked statins, while 19.2% stored non-statin lipid-lowering drugs. The poor drug availability was particularly serious in rural medical institutions (129). The PURE study evaluated the usage of secondary prevention drugs for patients with CVD in communities of countries with different income levels (133). Results signified a positive relation between the rates of use for statins and the economic level of country. In addition, the use rate of statins in patients with coronary heart disease or stroke in China was the lowest (1.7%) among all secondary prevention drugs, which is lower than the level in North America, Europe, the Middle East, South America, Malaysia, and South Asia and only slightly higher than the level in Africa.

Consequently, at the first step, we suggested to determine a patient journey of dyslipidemia with clear process and easy understanding and operation. Second, by improving the allocation of manpower and resources in primary medical institutions, it may help in making up for their vacancy in the management of dyslipidemia and gradually play their role in patient screening, initial diagnosis, referral, and follow-up management. Finally, a comprehensive management system should be established to prevent and control various cardiovascular diseases and their risk factors.

The quality of the studies included in this systematic review is generally high; consequently, the results of sensitivity analysis revealed that when the lowest score studies were excluded, only a slight impact on the results was achieved. Data from multiple provinces in Chinese Mainland with a large sample size was incorporated in our meta-analysis. However, some limitations in this study are shown as well. Firstly, due to limitations in data availability, the relationship between the prevalence of dyslipidemia and some factors cannot be explored by subgroup analyses and meta-regression. Therefore, this study is limited in exploring the influencing factors of dyslipidemia. Secondly, only epidemiological studies were included in our systematic review and meta-analysis, and the high degree of heterogeneity between studies is inevitable (134). However, subgroup analysis and meta-regression alleviate this issue to some extent. Finally, we cannot infer causality between dyslipidemia and other factors due to the cross-sectional design in all included studies.

## 5. Conclusion

Nearly half of Chinese adults suffered from dyslipidemia, while the most prevalent type of dyslipidemia was low levels of high-density lipoprotein cholesterol. Males and urban residents had a higher prevalence of dyslipidemia than their counterparts. This study further suggested extremely low rates of awareness, treatment, and control for dyslipidemia in Chinese adults. The government should increase the financial and manpower support for primary medical institutions and implement effective programs to prevent and control dyslipidemia.

## Data Availability

The original contributions presented in the study are included in the article/**Supplementary Material**, and further inquiries can be directed to the corresponding author.
